# Reactive Oxygen Species-Regulated Conjugates Based on Poly(jasmine) Lactone for Simultaneous Delivery of Doxorubicin and Docetaxel

**DOI:** 10.3390/pharmaceutics16091164

**Published:** 2024-09-03

**Authors:** Jyoti Verma, Vishal Kumar, Carl-Eric Wilen, Jessica M. Rosenholm, Kuldeep K. Bansal

**Affiliations:** 1 Pharmaceutical Sciences Laboratory, Faculty of Science and Engineering Åbo Akademi University, Biocity, Tykistökatu 6A, 20520 Turku, Finland; jyoti.verma@abo.fi (J.V.); vishal.kumar@abo.fi (V.K.); jessica.rosenholm@abo.fi (J.M.R.); 2 Laboratory of Molecular Science and Engineering, Faculty of Science and Engineering, Åbo Akademi University, Aurum, Henrikinkatu 2, 20500 Turku, Finland; carl-eric.wilen@abo.fi

**Keywords:** stimuli responsive, ROS, polymer–drug conjugate, targeted drug delivery, poly(jasmine lactone)

## Abstract

In cancer therapy, it is essential to selectively release cytotoxic agents into the tumor to prevent the adverse effects associated with anticancer drugs. Thus, in this study, a stimuli-sensitive polymer–drug conjugate was synthesized for selective drug release. Doxorubicin (DOX) and docetaxel (DTX) were conjugated onto novel poly(jasmine lactone) based copolymer via a thioketal (TK) linker. In addition, a photosensitizer (chlorin e6) was attached to the polymer, which served as a reactive oxygen species generator to cleave the TK linker. The conjugate is readily self-assembled into micelles less than 100 nm in size. Micelles demonstrate a notable increase in their ability to cause cell death when exposed to near-infrared (NIR) light on MDA-MB-231 breast cancer cells. The increase in cytotoxicity is higher than that observed with the combination of free DOX and DTX. The accumulation of DOX in the nucleus after release from the micelles (laser irradiation) was also confirmed by confocal microscopy. In the absence of light, micelles did not show any toxicity while the free drugs were found toxic irrespective of the light exposure. The obtained results suggest the targeted drug delivery potential of micelles regulated by the external stimuli, i.e., NIR light.

## 1. Introduction

In recent years, massive attempts have been made to develop nanosized drug delivery carriers (NCs) capable of effectively delivering drugs precisely to diseased areas. The major goals of NC-based cancer therapy include minimizing drug leakage in off-target areas and stimulating sufficient drug release within the tumor. However, in most cases, premature drug release from NCs diminished their advantages. Polymer–drug conjugates are capable of inhibiting premature drug release in cancer therapy, thereby ensuring minimal side effects caused by chemotherapeutic agents [1]. However, their widespread use is limited due to their non-selectiveness and slow drug release, leading to poor efficacy. Nevertheless, the incorporation of stimulus-sensitive characteristics in NCs could be a potential solution, enabling near-complete drug release in response to a stimulus associated with a specific disease symptom [2,3,4]. Conversely, endogenous stimuli (such as redox potential) can significantly vary from tumor to tumor because of the heterogeneity of cancer; thus, not all patients respond equally to the same therapy [5,6]. 

Thus, combining endogenous signals, such as reactive oxygen species (ROS), with exogenous signals, such as light, provides excellent control over targeted drug release due to non-dependency on a single stimulus [7,8]. In the past few years, chemotherapy in combination with photodynamic therapy (PDT) [9,10] has been used for concurrent cancer treatment. PDT is a method that uses photochemistry to induce cell death by producing ROS [11]. This is achieved by using a light-sensitive chemical photosensitizer and exposing it to light of a certain wavelength. Chlorin e6 (Ce6) is a frequently utilized photosensitizer in cancer treatment research and is an FDA-approved second-generation photosensitizer [12]. It is recognized for its capacity to generate significant levels of ROS; however, its practical use has been restricted owing to its poor water solubility and insufficient ability to specifically target diseased areas [13,14]. Thus, combining chemotherapy and PDT in stimuli-responsive NCs could be an improved approach to gain advantages of both therapies by alleviating the disadvantages associated with a single approach. 

For instance, Li et al. synthesized a small-sized nanocomposite containing Ce6-integrated gold nanoclusters for biological fluorescence imaging and photodynamic therapy. The nanocomposite demonstrated remarkable cancer cell mortality and the synergistic effect of gambogic acid via the combination of gold nanostars (AuNS) mediated mild photothermal therapy (PTT) and TCPP-mediated PDT [15]. In another study, Pei et al. used a ROS-activatable thioketal (TK) linker to conjugate doxorubicin (DOX) on polyphosphoester (PPE-TK-DOX). Later, Ce6 was encapsulated in NCs to give Ce6@PPE-TK-DOX, which effectively promotes localized ROS formation, breaking the TK linker and allowing tumor-specific drug administration with excellent light controllability using 660 nm light. However, encapsulating Ce6 in NCs did not prevent its premature release before reaching the target site, potentially diminishing the effectiveness of this approach [16,17]. Moreover, the administration of two chemotherapeutic drugs is beneficial in achieving enhanced therapeutic efficacy in cancer therapy. For instance, Xie et al. developed a thermosensitive hydrogel loaded with DOX and docetaxel (DTX) for synergistic chemotherapy. The therapeutic efficacy of co-delivering DOX and DTX nanoparticles was significantly enhanced (≈8-fold to that of DOX hydrogel), targeting triple-negative breast cancer cell lines. In vivo results demonstrated that dual-drug-loaded magnetic hydrogels showed greater anticancer effectiveness in reducing tumor size than single drug-loaded hydrogel [18].

Thus, in the present study, we synthesized stimuli-responsive NCs to deliver DOX and DTX simultaneously in a controlled manner at the tumor region. Both drugs were modified first with TK linker and later conjugated to the hydroxy-terminated poly(jasmine lactone) copolymer (Schemes S1 and S2) [19]. Poly(jasmine lactone) copolymer, due to its multifunctionality, offers the advantage of introducing numerous functional groups of interest, which can be utilized to conjugate different molecules [20]. It has been reported that the TK linker is readily cleaved in the presence of abundant ROS. The same claims have also been made for disulfide linkers in reductive environments, but in our previous study, we failed to observe triggered/complete release of conjugated drugs owing to the core-corona structure of micelles where the linker and drug were hidden within the hydrophobic regions [21]. Thus, in this study, Ce6 was also conjugated to the polymer as an additional ROS generator to avoid the incomplete release of drugs and premature release of Ce6. 

## 2. Methods

### 2.1. Synthesis of Thioketal Linker

Thioketal linker was synthesized following a reported procedure [16]. Briefly, dry hydrogen chloride gas was mixed with 5.8 g of acetone (98.2 mmol) and 5.2 g of 3-mercaptopropionic acid (49.1 mmol) in a flask. The mixture was then stirred at room temperature for 6 h. After 6 h of stirring, the reaction mixture was placed on an ice–salt mixture for crystallization of the product. The crystallized product was washed several times with cold hexane and ice-cold water. The white-colored thioketal linker was then dried in a vacuum and characterized by ^1^HNMR (yield-3.6 g) (Figure S1). ^1^H NMR (500 MHz, CDCl_3_) δ 2.91 (t, 4H), 2.68 (t, 4H), 1.60 (s, 6H).

### 2.2. Conjugation of Doxorubicin to Thioketal (DOX-TK)

Thioketal linker (0.07 g, 0.28 mmol), EDC (0.064 g, 0.33 mmol), and NHS (0.038 g, 0.33 mmol) were dissolved in 10 mL of DCM and stirred for 1 h at 0 °C. In a separate flask, doxorubicin hydrochloride (DOX, 0.15 g, 0.28 mmol) (see Figure S2 for ^1^HNMR) was dissolved in 10 mL of DCM containing 100 µL of triethylamine and stirred for 1 h before being transferred to the flask containing TK linker. The reaction mixture was further stirred in the dark, and monitored by TLC (30:70 MeOH:DCM). After 24 h, the reaction mixture was filtered, and the solvent was evaporated and used in the next step without purification.

### 2.3. Conjugation of Docetaxel to Thioketal (DTX-TK)

Thioketal linker (0.047 g, 0.18 mmol) and EDC (0.069 g, 0.36 mmol) were dissolved in a flask containing 10 mL of DCM and stirred for 30 min at 0 °C. Docetaxel (0.15 g, 0.18 mmol) (see Figure S3 for ^1^HNMR) and DMAP (0.044 g, 0.36 mmol) were dissolved separately in DCM and added to the above-prepared solution and stirred for 36 h in a dark environment. The reaction mixture was monitored by TLC (30:70 MeOH:DCM). The reaction mixture was filtered, the solvent was evaporated, and the dry product was used in the next step without further purification.

### 2.4. Synthesis of Functionalized Block Co-Polymer of Poly(jasmine lactone), i.e., mPEG-b-PJL-OH

The block copolymer of poly(jasmine lactone) and its functionalization was performed according to the previously reported procedure and the same polymer is used in this study [21]. Briefly, the amphiphilic block copolymer mPEG-b-PJL was synthesized by ring-opening polymerization at 50 °C using methoxy (polyethylene glycol) (mPEG_5K_) as an initiator and TBD as a catalyst. Dried mPEG_5K_ (5 g, 1.0 mmol) was added to a flask containing dry jasmine lactone (5.04 g, 30.0 mmol), and the mixture was heated to 50 °C and stirred for 10 min, to obtain a homogeneous mixture. TBD (0.11 g, 0.8 mmol) was added, and the mixture was allowed to stir for 7 h at 50 °C (88% conversion as per ^1^HNMR). The reaction mixture was then cooled, and quenched by adding benzoic acid (0.3 g) solution in acetone (5 mL). The resulting polymer was precipitated in cold methanol followed by the removal of residual solvent in a vacuum. 

The copolymer mPEG-b-PJL was recovered as wax-like material with 81.7% yield (8.2 g, 0.9 mmol, % yield in moles—90%) and functionalized by thiol-ene click reaction. In short, mPEG-b-PJL (1.0 g, 0.11 mmol) and 2-mercaptoethanol (0.8 g, 10.1 mmol) were dissolved in DCM (5 mL). 2,2-Dimethoxy-2-phenylacetophenone (DMPA, 0.097 g, 0.38 mmol) was then added to the above mixture and stirred for 4 h in a UV cabinet fitted with a blacklight 368 nm lamp (15 W, Sylvania, Vantaa, Finland). The reaction mixture was then precipitated in cold diethyl ether, followed by the removal of residual solvent in vacuum to obtain the product, which was a white sticky solid with 94% yield (1.11 g, 0.10 mmol, % yield in moles—90%). ^1^HNMR was used to measure the molecular weight of the functionalized polymer by comparing the number of protons of thiol-substituted PJL ester linkage at 4.9 ppm with respect to the protons of the initiator (mPEG) at 3.3 and the protons adjacent to the pendant chain of PJL at 0.98 ppm. The splitting of the methyl peak at 0.98 ppm (position N) is due to the random attachment of thiol on the mPEG-b-PJL pendent chain (Figure S4).

Calculated molecular weight of mPEG-b-PJL-OH by ^1^HNMR (based on 100% conversion of mPEG-b-PJL into mPEG-b-PJL-OH)—10.5 kDa.

^1^H NMR (500 MHz, CDCl_3_) mPEG-b-PJL-OH, δ 5.31–4.80 (m, 23H), 4.24 (m, 2H), 4.08 (s, 1H), 3.72 (m, 45H), 3.69–3.48 (s, 506H), 3.40 (s, 3H), 2.71 (m, 44H), 2.58 (m, 21H), 2.35 (s, 46H), 1.80–1.36 (m, 258H), 0.98 (m, 70H).

### 2.5. Conjugation of Ce6, DOX-TK, and DXT-TK to mPEG-b-PJL-OH

Ce6 (0.066 g, 0.11 mmol) and EDC (0.043 g, 0.22 mmol) were dissolved in a flask containing 10 mL of DMF and separately, mPEG-b-PJL-OH (0.5 g, 0.056 mmol) and DMAP (0.014 g, 0.11 mmol) were dissolved in a falcon tube containing DMF and added to the above reaction mixture and stirred on an ice bath in the dark. After 12 h of stirring, the reaction was monitored by TLC (30:70 MeOH:DCM), which suggests complete consumption of Ce6. 

Synthesized intermediates DOX-TK, DTX-TK, and EDC (0.04 g, 0.2 mmol) in 10 mL of DMF were added at 0 °C and stirred for 30 min, followed by addition to the above reaction mixture. DMAP (0.007 g, 0.056 mmol) was added to the reaction mixture, and stirring continued. The progress of the reaction was monitored by TLC (in solvent 20:80 MeOH:DCM). After the consumption of the starting materials, the reaction mixture was transferred to a dialysis tube (MWCO—2 kDa) and dialyzed for two weeks in water to remove unreacted reactants and reagents. The purified solution was then freeze-dried to afford PJL-DOX-DTX. To calculate the molecular weight of polymers and their conjugate via ^1^HNMR, the number of protons of the thiol-substituted PJL ester linkage at 4.9 ppm was compared with the number of protons of the initiator (mPEG) at 3.3 ppm and the protons adjacent to the pendant chain of PJL at 0.98 ppm. Moles of DOX, DTX, and Ce6 conjugated to polymer were measured by comparing the number of aromatic protons at 7 to 8 ppm and the hydroxyl protons of DOX at 14.5 ppm 13.2 ppm and for Ce6 at 13.92 ppm, with respect to the protons of the initiator (mPEG) at 3.3 ppm (Figure S5).

Calculated molecular weight by ^1^HNMR—14.7 kDa.

^1^H NMR (500 MHz, DMSO-d6) δ 14.05 (s, 2H), 13.92 (s, 1H), 13.29 (s, 2H), 13.18–13.13 (s, 1H), 8.00–7.10 (m, 68H), 6.33 (m, 1H), 4.82 (m, 23H), 3.23 (s, 3H), 1.05–0.71 (m, 73H).

### 2.6. Preparation of Polymeric Micelles

Micelles of the conjugate were prepared by the nanoprecipitation method [22,23], in which polymer–drug conjugates (15 mg) were dissolved in acetone (3 mL) with the help of a vortex and added dropwise into the PBS (5 mL) and stirred for at least 12 h at room temperature to ensure the complete removal of the organic solvent (acetone). To separate the precipitated polymer, if any, micelles were centrifuged for 10 min at 13,500 RPM (Microcentrifuge Scanspeed, Labogene, Lynge, Denmark). Next, the supernatant was filtered using a 0.45 µm polypropylene membrane filter. 

Similarly, Ce6 micelles were prepared by using mPEG-b-PJL-OH polymer (20 mg) and Ce6 (1.2 mg), which was dissolved in ethanol (1 mL) and added dropwise into the milli-Q water (1 mL). 

### 2.7. Characterization of Micelles

The drug concentrations were measured using a UV-Vis spectrophotometer (NanoDrop 2000c from Thermo Fisher Scientific, Vantaa, Finland) after diluting the micelles in MilliQ water at a wavelength of 481 nm for DOX and at 663 nm for Ce6 (Figure S6). The size and polydispersity index of PJL-DOX-DTX micelles were determined using dynamic light scattering (DLS) on a ZetaSizer Nano-ZS instrument (Malvern Instruments, Malvern, UK) at 25 °C. After diluting the micelle to a concentration of 300 µg/mL (equivalent to polymer) with MilliQ water, the samples were placed in the appropriate cuvettes for analysis. 

TEM images were obtained using a JEM 1400-Plus (JEOL Ltd., Tokyo, Japan) microscope to confirm the size and to determine the shape of formulated micelles. One drop of PJL-DOX-DTX micelles solution in miliQ water (typically 300 μg mL^−1^) was dropped onto a copper grid and allowed to dry in air. Samples were then imaged after staining with uranyl acetate.

### 2.8. In Vitro Reactive Oxygen Species (^1^O_2_) Generation and TK Linker Cleavage

DPBF (1,3-Diphenylisobenzofuran) was often utilized to determine the ^1^O_2_ generation due to its highly reactive nature with singlet oxygen [24]. Samples of free Ce6 and PJL-DOX-DTX micelles (equivalent to free Ce6, as per UV) were prepared in DMSO and water, respectively, and divided into four vials and assigned as (1) free Ce6; (2) free Ce6 + laser (665 nm, 0.5 W cm^−2^); (3) PJL-DOX-DTX micelles; and (4) PJL-DOX-DTX micelles + laser (665 nm, 0.5 W cm^−2^). The experiment was performed in two setups: in setup-1, the DPBF was added beforehand to the above vials, while in setup-2, the DPBF was added after the laser irradiation to the above solutions in the dark. In setup 1, after 0, 30, 60, 120, 240, and 300 s of sample treatments (with and without laser irradiation), the UV-Vis measurements (410 nm) were performed [25,26]. In setup 2, after 0, 30, 60, 120, 240, and 300 s of sample treatments (with and without laser irradiation), DPBF was added to all samples and incubated for 5 min before UV measurements. 

To detect the TK linker cleavage by NMR, the thioketal linker and Ce6 were added into the NMR tube, and spectra were taken in DMSO d6. The tube was then irradiated by near-infrared red (NIR) light for 15 min and measured again by NMR. Spectra before and after laser irradiation were analyzed to detect the signals of degraded product from the thioketal linker.

### 2.9. Cell Studies

#### 2.9.1. Cell Media

The human triple-negative breast cancer cell line MDA-MB-231 cells (ATCC, Manassas, VA, USA) were cultured in high glucose Dulbecco’s modified Eagle’s medium (DMEM) supplemented with 10% heat-inactivated fetal bovine serum (FBS), 2 Mm L-glutamine, 100 IU/mL penicillin, and 100 µg/mL streptomycin (all from Lonza) at 37 °C with 5% CO_2_. Cells were subcultured after they achieved 80–90% confluency. 

#### 2.9.2. Cell Cytotoxicity Study

The cell growth inhibition ability of samples were tested on MDA-MB-231 cells via Alamar Blue cell viability assay, using a previously published method with slight adjustments [27]. A total of 100 µL of cell stock suspension with a concentration of 70,000 cells/mL was placed in a 96-well plate and left to incubate for 24 h. All samples were prepared in concentrations of 400, 600, and 800 ng/mL (with respect to DOX), except for Ce6, which had concentrations of 240, 360, and 480 ng/mL. The samples were prepared from stocks in growth media prewarmed to 37 °C. After 24 h, the cell media in a 96-well plate was replaced with samples and incubated at 37 °C with 5% CO_2_. The laser light treatment was conducted in two experimental setups: in setup 1, the cell medium was replaced with fresh media after 4 h of incubation, while in setup 2, no changes were made. After 4 h, samples were irradiated for 2 min with NIR laser (650 nm, 0.5 W cm^−2^) and incubated for another 44 h (total 48 h). After 48 h incubation, 10 µL of Alamar Blue cell proliferation reagent was added to the wells and incubated for an additional 3 h. The samples’ fluorescence was captured following the manufacturer’s protocol, with excitation at 540 nm and emission at 560–590 nm. The cell proliferation percentage was measured in comparison to untreated cells, which were considered to have 100% viability. Appropriate controls were used to determine the cell viability. A comparable method was used to assess the impact of drug release in the absence of NIR light. A similar method was used to perform the cytotoxicity assay on Vero H10 cells with the concentration range of 2.5, 5, and 10 µg/mL (with respect to DOX).

### 2.10. Intracellular Generation of ROS

In order to identify the production of ROS, MDA-MB-231 cells were seeded in 48-well plates with a density of 20,000 cells per well in DMEM high glucose media. Following a 24 h incubation at 37 °C, the media was removed and cells were treated with 3 different samples, i.e., (a) Ce6 micelles (400 ng/mL equivalent to Ce6) + DCFH-DA (50 µM), (b) free Ce6 (400 μg/mL) + DCFH-DA (50 µM) and (c) DCFH-DA (50 µM) only in cell culture media and incubated for 3 h. The stock solutions of free Ce6 and DCFH-DA were prepared in DMSO. The cells were then irradiated with a 650 nm laser at a power density of 0.5 W cm^−2^ for 3 min after replacing the culture media with fresh media. Subsequently, the cells were incubated again for 15 min at 37 °C. The media was removed, cells were washed with PBS and fresh PBS was added before fluorescence measurement at 527 nm (λ_ex_) and 485 nm (λ_em_) [28,29]. 

### 2.11. Confocal Laser Scanning Microscopy (CLSM)

For CLSM observation, MDA-MB-231 cells were cultured on autoclaved coverslips (22 mm × 22 mm) in 6-well plates with a concentration of 70,000 cells/mL and incubated overnight for the attachment. The following day, cells were treated with PJL-DOX-DTX micelles (400 ng/mL equivalent to DOX) in cell media for 12 h at 37 °C with 5% CO_2_. Following incubation, cells were washed with PBS to remove non-internalized micelles, media was replaced, and one sample was irradiated with 650 nm laser at the power of 0.5 W cm^−2^ for 3 min and further incubated for 15 min. After incubation, cells were rinsed with PBS, fixed with 4% PFA for 10 min at room temperature, and washed thrice with PBS followed by a final washing with MiliQ water. Coverslips were mounted with VECTASHIELD mounting medium with DAPI, and then observed by CLSM (3i CSU-W1 spinning disk) with SlideBook 6 software using red (561 nm) and blue (405 nm) filters [27].

### 2.12. Statistical Analysis

Statistical analysis was performed using one-way ANOVA with Tukey’s multiple comparisons test, with a significance threshold set at *p* < 0.05 (95% confidence interval) unless stated otherwise. Statistical analysis was conducted using GraphPad Prism software (version 6) with a sample size of n = 3. Statistical significance levels are categorized as follows: extremely significant (****, *p* < 0.0001), extremely significant (***, *p* = 0.0001 to 0.001), very significant (**, *p* = 0.001 to 0.01), significant (*, *p* = 0.01 to 0.05), and not significant (ns, *p* ≥ 0.05).

## 3. Results and Discussion

**Scheme 1:**
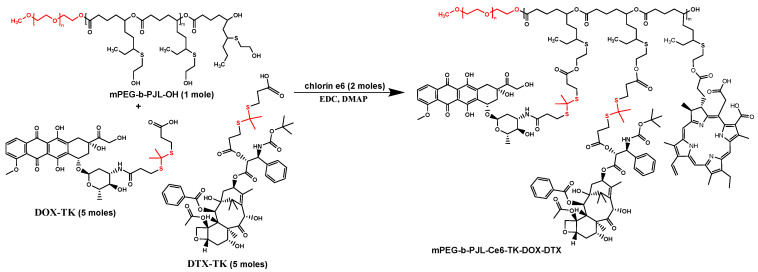
Conjugation of doxorubicin, docetaxel, and chlorin e6 to the mPEG-b-PJL-OH polymer to obtain mPEG-b-PJL-Ce6-TK-DOX-DTX (PJL-DOX-DTX). DOX-TK and DTX-TK were synthesized separately before addition to the reaction. (N-(3-Dimethylaminopropyl)-N′-ethylcarbodiimide (EDC), 4-(Dimethylamino) pyridine (DMAP).

To construct an oxidation-sensitive polymer–drug conjugate, we attached TK derivatives of DOX and DTX to mPEG-b-PJL-OH using a simple carbodiimide coupling reaction. The mPEG-b-PJL-OH was first reacted with Ce6, and later DOX-TK and DTX-TK were added to this solution to obtain mPEG-b-PJL-Ce6-TK-DOX-DTX, represented as PJL-DOX-DTX from this point onwards (Scheme 1). The progress of the reaction was followed by TLC, and the product was purified by dialysis after consumption of all starting materials. We were aware that TK derivatization of DOX and DTX is not selective, and it is possible that both acid functionality of the TK linker can be capped by the drug, but the undesired product was easily removed during dialysis due to a significant difference in molecular weight. The sample was then run into HPLC to ascertain the absence of free drug molecules in the product. HPLC traces of pure DOX, DTX, and Ce6 showed retention times of 6.56, 15.49, and 17.84 min, respectively, whereas these peaks were absent in the trace of PJL-DOX-DTX, revealing the absence of starting materials in the final purified product (Figure S7). 

The cleavage of the thioketal linker in the presence of ROS generated by Ce6 upon NIR light irradiation was assessed by the ^1^HNMR spectroscopy. After irradiation of the sample, the appearance of signals (Figure S8) at 2.09 ppm, 2.65 ppm, and 2.9 ppm (acetone and 3-mercaptopropionic acid, respectively) suggested the oxidative cleavage of the TK linker. Upon oxidation, the TK linker was converted into starting material, i.e., 3-mercaptopropionic acid and acetone. An additional peak at 1.9 ppm was also observed, which could belong to the intermediate product during the oxidation of the TK linker as reported by Thayumanavan and coworkers [30].

Additionally, ^1^HNMR was used to determine the molar mass of the polymer and the conjugation efficiency of DOX, DTX, and Ce6. The molecular weight of mPEG-b-PJL-OH and PJL-DOX-DTX (Figures S4 and S5) were calculated from ^1^HNMR spectra and found to be 10.5 kDa, and 16.5 kDa, respectively, by comparing the peak positions of protons from 7.1 to 8.0 ppm of both DOX and DTX, hydroxyl proton of DOX (D, E) at 14.05 and 13.29 ppm and protons of Ce6 (F, G and H) at 13.92, 13.18, and 6.3 ppm in respect to methylene protons (A, C) at 3.3 and 0.98 ppm of mPEG-b-PJL-OH. Based on these calculations, two molecules of DOX, four molecules of DTX, and one molecule of Ce6 were attached to mPEG-b-PJL-OH. This molar ratio suggests 6.58, 19.56, and 3.61 wt% of DOX, DTX, and Ce6, respectively, conjugated to the polymer. 

**Figure 1:**
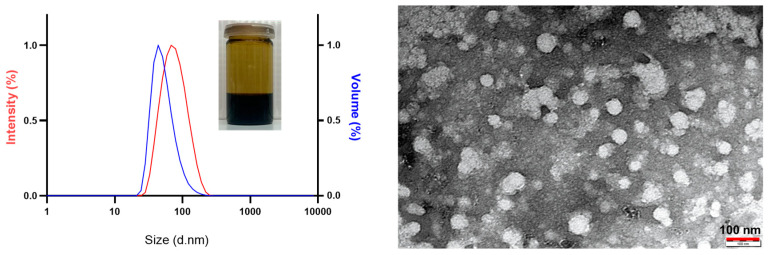
Particle size distribution by volume and intensity percentage determined by DLS and TEM image to confirm size and shape of micelles (300 µg/mL concentration). Inset: picture of the formulation.

Further, we attempted to utilize HPLC to calculate the amount of DOX and DTX but realized that cleavage attempts of the TK linker using H_2_O_2_ led to drug degradation (performed on pure drugs to understand the issue). We recognize the complexity of the system and understand that an alternative method with highly sophisticated analytical instruments is required to calculate accurate amounts of drugs conjugated onto the polymer. Nevertheless, our primary goal is to establish the advantage of dual drug delivery in cancer therapy by precisely controlling the release of drugs; thus, we move forward by considering the tentative drug quantities calculated by ^1^HNMR and UV-Vis. The micelles of PJL-DOX-DTX were prepared using an industrial-friendly nanoprecipitation method and then characterized for size and Ce6 content. The UV estimation of micelles suggests the presence of 7.33 wt% (220 µg/mL) of Ce6, and 12.37 wt% (371 µg/mL) of DOX (approx.) with a Z-average size of ≈72 nm and PDI of 0.166 (Figure 1). However, the amount of DOX calculated by UV is not accurate due to the overlapping of the Ce6 peak at 481 nm (Figure S6). The content of Ce6 found in mPEG-b-PJL-OH micelles was 1.08 mg/mL using UV-Vis spectroscopy at λ_max_ 663 nm.

**Figure 2:**
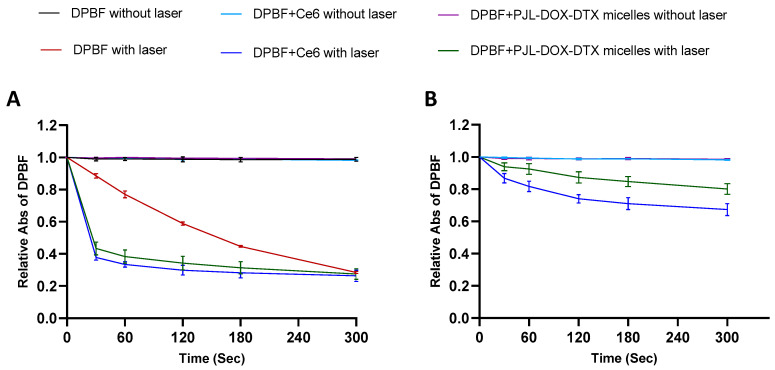
(**A**) Absorbance changes of DPBF at wavelength 410 nm with DPBF added before the laser irradiation (**B**) Absorbance changes of DPBF at wavelength 410 nm with DBPF added after the laser irradiation (n = 3).

To verify the ability of the PJL-DOX-DTX micelles to generate ^1^O_2_, UV-Vis tests were performed with two different setups. In Figure 2A, with setup-1, where DPBF was added along with polymer and Ce6, the reduction in the absorbance of DBPF suggested the generation of ROS. However, we also performed a control experiment, where DPBF alone was irradiated with a laser, and surprisingly, a similar reduction in its absorbance was observed in the absence of Ce6. These results reveal that DPBF was not stable in the NIR light irrespective of the presence of photosensitizer, and consequently the results obtained are not reliable. This leads us to modify the reported procedure, and thus an experiment was designed as setup 2 where DPBF was added after the laser irradiation to protect its degradation by NIR light. The results in Figure 2B indicate the generation of ROS by Ce6 either in free form or conjugated with the polymer, as evidenced by the reduction in absorbance of DBPF in the irradiated samples.

It is expected that due to the overproduction of ROS in tumor regions, the TK linker of PJL-DOX-DTX will be dissociated, releasing the free drug to exert its pharmacological action. On the other hand, low levels of ROS in healthy tissues are not capable of ripping the bond, and thus, normal cells remain unaffected. The efficiency of this system can be induced externally by applying NIR light, which in turn generates ROS due to the presence of photosensitizer [26,31]. Thus, to evaluate the controlled release in the presence or absence of external stimuli (NIR light), we performed cytotoxicity assays in the presence or absence of 650 nm NIR laser (0.5 W cm^−2^). Free DOX, DTX, Ce6, and PJL-DOX-DTX with equivalent concentrations of Ce6 (240, 360,480 ng mL^−1^) in cell media were incubated with MDA-MB-231 cells for 48 h. According to the tentative UV results, the incubated concentration of PJL-DOX-DTX corresponds to DOX (400, 600, and 800 ng mL^−1^). The treatment was performed in two experimental settings. In one setup, cell media was replaced with fresh media after 4 h of incubation, whereas in another setup, the media was not changed.

The necessity for this experimental condition emerges because of our previous unsuccessful attempt, in which we observed no noticeable difference in cell death with or without light irradiation. In this experimental configuration, we began by exposing the PJL-DOX-DTX micelles in a glass vial to NIR light for 5 and 10 min (to cleave the TK linker) before incubating the cells. As expected from the free drugs, being highly toxic, DOX inhibited cell proliferation by ≈95% and DTX by ≈50% cells at 20 µg mL^−1^ concentration after 48 h incubation. Considering the poor aqueous solubility of DTX, no significant difference was observed in cell proliferation percentages at different concentrations of DTX [27] (Figure S9). Surprisingly, no significant change in the toxicity pattern was observed between laser-treated and untreated micelles. This leads us to speculate that the cleaved modified DOX and DTX structures may have been incapable of penetrating the cells; thus, no increment in cytotoxicity was observed (Figure S9 and Scheme S3). Thus, to understand this, we removed the media from one experimental setup to check if the cell proliferations remained the same compared with the samples, where the media was not replaced. 

**Figure 3:**
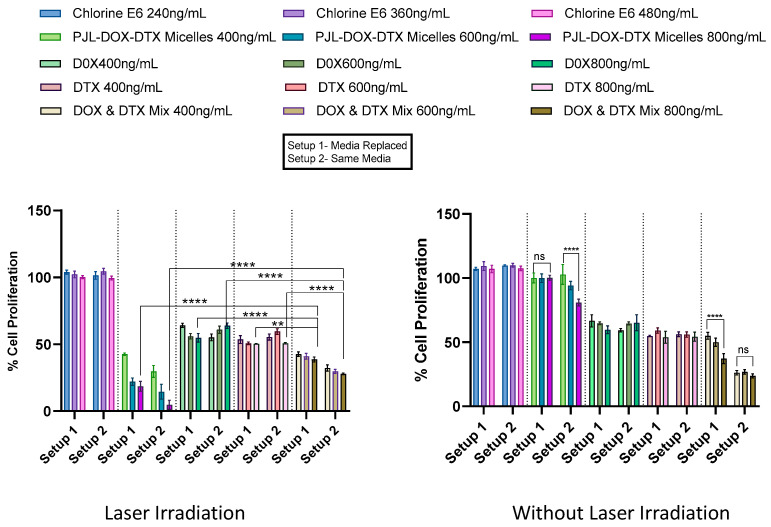
Percentage cell proliferation inhibition study of DOX, DTX, mixture of DOX-DTX, Ce6, and PJL-DOX-DTX micelles (equivalent to 240, 360, 480 ng mL^−1^ for Ce6) in the presence and absence of laser light with or without media replacement on MDA-MB-231 cell lines. ns, *p* ≥ 0.05, **, *p* < 0.01, ****, *p* < 0.0001.

When the cells were irradiated directly with NIR light, we observed no significant difference in cell proliferation inhibition by free DOX (≈35–45%) and DTX (≈40–45%) compared with samples without laser irradiation regardless of the experimental setup (Figure 3). However, the mixture of DOX-DTX was found to be more effective than individual drugs and demonstrated ≈60% cell growth inhibition due to the synergistic effect. Moreover, in setup 2, the DOX-DTX mixture was found to be highly effective with a cell growth inhibition of ≈75%. 

The combination index is a quantitative assessment of the synergy or antagonism of two or more drugs when administered together. The estimated combination index (CI) may be less than 1 (CI < 1), greater than 1 (CI > 1), or equal to 1 (CI = 1), indicating synergistic, antagonistic, and additive effects of the drug combination, respectively. The response additivity approach (CI = Ea + Eb/Eab), where Ea, Eb, and Eab indicate the effect of DOX, DTX, and combined DOX + DTX at 800 ng/mL concentration, respectively, on MDA-MB-231 cells (Figure 3, No laser irradiation setup 2) was used to calculate the combination index of DOX and DTX. The CI value calculated based on the formula is 1.05, which indicates the additive effect of the drugs [32]. 

No cytotoxicity was observed with Ce6 in any of the experimental settings, suggesting that the ROS produced by Ce6 taken up by the cells is not sufficient to elicit cell death after laser irradiation. The poor uptake of free Ce6 could be the reason for this phenomenon. However, nanoparticles were utilized to improve the cellular uptake of Ce6 where only 20–30% cell growth inhibition was observed on MDA-MB-231 cells at a concentration of 400 ng/mL [33]. Interestingly, the PJL-DOX-DTX micelles inhibited cell proliferation by up to ≈95% after light treatment, (much higher than Ce6 nanoparticles reported by [33]) suggesting that the ROS generated from the photosensitizer was sufficient to cleave the TK linker and thus enhance the cytotoxicity of these samples. In contrast, no cell growth inhibition was observed in the absence of laser light treatment (in setup 1), whereas approximately 20% inhibition was observed in setup 2.

On the basis of these results, we discovered higher efficacy in samples where the medium was not replaced (setup 2), disproving our assumption that modified drug structures could not penetrate the cells. However, we failed to hypothesize the reason behind the poor efficacy of samples toward cell death when irradiated outside the cells (Figure S9). In addition, treatments at concentrations above 800 ng mL^−1^ of PJL-DOX-DTX micelles in the presence of NIR light resulted in 100% cell death, whereas the higher concentrations of free drugs DOX and DTX only inhibited cell proliferation by ≈85% and ≈20%, respectively (Figure S10), suggesting the superiority of the developed formulation.

**Figure 4:**
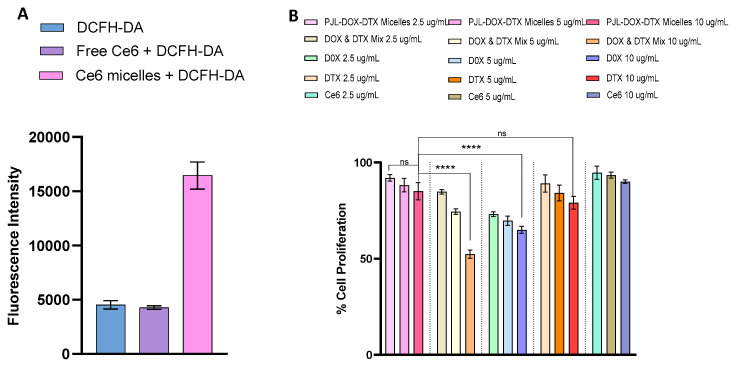
(**A**) DCFH-DA fluorescence intensity post treatment with Ce6 micelles and free Ce6 in MDA MB-231 cells after 3 h incubation and laser irradiation. (**B**) Percentage cell proliferation inhibition study of DOX, DTX, mixture of DOX-DTX, Ce6, and PJL-DOX-DTX micelles (2.5, 5, 10 μg mL^−1^ with respect to DOX) on Vero H10 cell lines after 48 h incubation. ns, *p* ≥ 0.05, ****, *p* < 0.0001.

To confirm the potential of Ce6 in generating ROS intracellularly, a DCFH-DA probe was used [21]. Free Ce6 and Ce6 encapsulated in micelles were used to determine the photosensitizing property. It is reported that the non-fluorescent DCFH-DA converted into fluorescent 2′-7′dichlorofluorescein (DCF), in the presence of ROS. As shown in Figure 4A, high fluorescence was observed in cells treated with Ce6 micelles after NIR laser irradiation. In contrast, no increment in fluorescence was observed in cells treated with free Ce6 compared to the control suggesting the poor uptake of free Ce6 by the cells and consequently low ROS production upon laser irradiation. The obtained results clearly demonstrated that Ce6 is able to produce ROS intracellularly upon laser irradiation. 

Since MDA-MD-231 are cancer cells, the presence of certain levels of intrinsic ROS results in the inhibition of cell proliferation by 20% at 800 ng mL^−1^ concentration without light irradiation. Thus, to prove the safety of our formulation (PJL-DOX-DTX micelles), an additional cytotoxicity experiment was performed on a normal cell line, i.e., Vero H10 cells, where intrinsic ROS production would be minimal. As presented in Figure 4B, PJL-DOX-DTX micelles show negligible cell death irrespective of the concentration used, whereas pure DOX, DTX, and a mixture of DOX and DTX at higher concentrations show 30%, 25%, and 45% cell proliferation inhibition, respectively, indicating the safety of our formulation on normal cell line. The poor efficacy of DOX to kill Vero H10 cells compared with cancer cells was also observed earlier, where free DOX was approximately 50% less effective on Vero cells compared with cancerous HepG2 cells [34]. Nevertheless, our formulation was found to be safer than the mixture of free DOX-DTX, suggesting its capability to reduce unwanted side effects.

**Figure 5:**
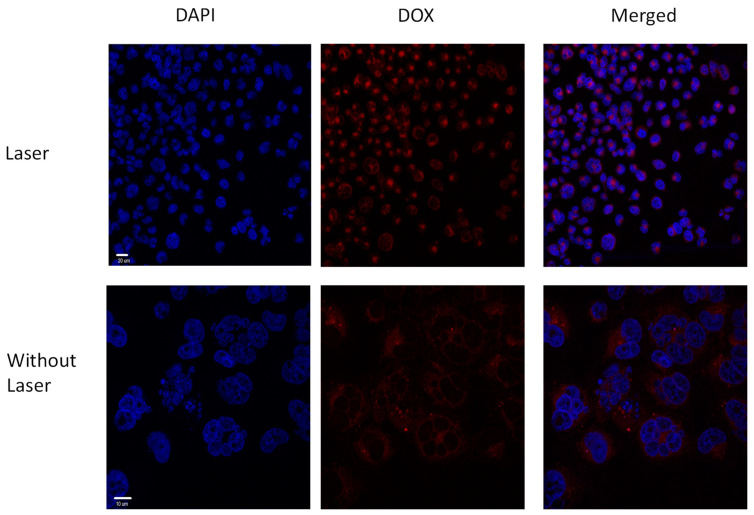
CLSM images of PJL-DOX-DTX micelles internalized in MDA-MB-231 cells after 12 h incubation with or without laser treatment. Cells were treated with 400 ng mL^−1^ equivalent concentration of DOX. Cell nucleus was stained with DAPI (blue), while DOX inherent red fluorescence was used to detect PJL-DOX-DTX micelles. Scale bar—20 µm (laser) and 10 µm (without laser).

Furthermore, the internalization of PJL-DOX-DTX micelles was investigated using CLSM by using the inherent fluorescent property of DOX. Two microscopy samples were prepared in which, one sample was irradiated with a laser to dissociate the DOX from the polymer. As shown in Figure 5, weak fluorescence of DOX was observed from non-irradiated samples compared to irradiated ones, suggesting the release of DOX after laser irradiation owing to high fluorescence signals. Strong red DOX fluorescent signals were observed in cell nuclei from laser irradiation samples suggesting the DOX localization in the nucleus (site of action) after release. In contrast, DOX was mainly localized in the cytoplasm in non-irradiated samples, indicating that DOX was still conjugated with polymer, resulting in poor cytotoxicity (Figure 3). These results demonstrated NIR laser-controlled DOX release upon cleavage of thioketal linkage, which efficiently generated free DOX concentration in cell nuclei. 

## 4. Conclusions

In this study, we successfully conjugated two anticancer drugs, i.e., DOX and DTX, via ROS-sensitive TK linker onto the free hydroxyl groups of poly(jasmine lactone) block copolymer to impart stimuli-sensitive drug release characteristics to the system. The photosensitizer Ce6 was additionally attached to the polymer as an additional source of ROS to accelerate drug cleavage in the presence of NIR light. The generation of ROS from Ce6 upon NIR irradiation was successfully established by in vitro experiments. The PJL-DOX-DTX conjugate is readily self-assembled into sub-100 nm micelles in the aqueous phase. In vitro cytotoxicity assay on MDA-MB-231 cells suggested NIR light-driven drug release from the micelles, reflecting the higher control on the cytotoxic action of chemotherapeutic drugs and thus, consequently, could be beneficial in reducing the adverse effects. The NIR-regulated release of DOX was successfully established through CLSM. Further, higher cytotoxicity was observed from micelles compared with free drugs in cancer cells, whereas no toxicity was observed from micelles on normal cells in the absence of light, clearly indicating the potential of the developed formulation in reducing side effects. However, because of the complexity of the proposed system, basic analytical tools failed to determine the exact amount of drug loading in PJL-DOX-DTX micelles. Thus, our future work will focus on developing analytical methods and approaches to accurately calculate drug content.

## Data Availability

Materials, instruments, characterization, and other relevant data are available as Electronic Supplementary Information (ESI).

## Supplementary Materials

The following are available online at https://www.mdpi.com/article/10.3390/pharmaceutics16091164/s1, Scheme S1: Synthesis of mPEG-b-PJL and its functionalization into mPEG-b-PJL-OH; Scheme S2: (A) Synthesis of thioketal linker (B) conjugation of thioketal linker to Doxorubicin and (C) conjugation of thioketal linker to Docetaxel; Scheme S3: Cleavage of thioketal bond in presence of reactive oxygen species (ROS) leading to the release of modified DOX and DTX structure [35]; Figure S1: ^1^HNMR spectra of synthesized thioketal linker in CDCl_3_; Figure S2: ^1^HNMR spectra of doxorubicin in DMSO-d_6_; Figure S3: ^1^HNMR spectra of docetaxel in DMSO-d_6_; Figure S4: ^1^HNMR spectra of mPEG-b-PJL-OH in CDCl_3_. Molecular weight was calculated by comparing the protons at 4.9 ppm with the protons of mPEG at 3.3; Figure S5: ^1^HNMR spectra of PJL-DOX-DTX in DMSO-d6; Figure S6: UV-Vis spectra of pure DOX, PJL-DOX-DTX micelles in milliQ water, and Ce6 in ethanol; Figure S7: HPLC traces of (A) doxorubicin (B) docetaxel, (C) chlorin e6 and (D) PJL-DOX-DTX; Figure S8: Cleavage of thioketal linker in the presence of Ce6. (A) ^1^HNMR after irridiation detects the signals at 2.09 ppm, 2.65 ppm, and 2.9 ppm, which belongs to the degraded product (acetone and 3-mercaptopropionic acid respectively) of thioketal linker. (B) ^1^HNMR before irridiation; Figure S9: Cytotoxicity study of free DOX, DTX and PJL-DOX-DTX micelles with light expsoure before incubation on MDA-MB-231 cell lines for 48 h; Figure S10: Cytotoxicity study of free DOX, DTX and PJL-DOX-DTX micelles at higher concentration on MDA-MB-231 cell lines for 48 h.
