# Supplementary material for: Reactive Oxygen Species-Regulated Conjugates Based on Poly(jasmine) Lactone for Simultaneous Delivery of Doxorubicin and Docetaxel
Source: Pharmaceutics. 2024 Sep 3;16(9):1164. doi: 10.3390/pharmaceutics16091164 (PMC11434831; doi:10.3390/pharmaceutics16091164)
Supplement: Supplementary file 1 [file pharmaceutics-16-01164-s001.zip › pharmaceutics-3164033-supplementary.pdf]

Electronic Supplementary Information (ESI)

Reactive Oxygen Species-Regulated Conjugates Based on Poly(jasmine) Lactone for Simultaneous Delivery of Doxorubicin and Docetaxel

Jyoti Verma [1], Vishal Kumar [1,2], Carl-Eric Wilén [2], Jessica M. Rosenholm [1] and Kuldeep K. Bansal [1,2,*]

[1] Pharmaceutical Sciences laboratory, Faculty of Science and Engineering, Åbo Akademi University, Biocity, Tykistökatu 6A, 20520 Turku, Finland; jyoti.verma@abo.fi (J.V.); vishal.kumar@abo.fi (V.K.); jessica.rosenholm@abo.fi (J.M.R.)

[2] Laboratory of Molecular Science and Engineering, Faculty of Science and Engineering, Åbo Akademi University, Aurum, Henrikinkatu 2, 20500 Turku, Finland; carl-eric.wilen@abo.fi

* Correspondence: kuldeep.bansal@abo.fi

Table of Contents

Materials: 2

Instruments: 2

Nuclear Magnetic Resonance (NMR) spectroscopy: 2

High Performance Liquid Chromatography (HPLC): 2

Scheme S1. Synthesis of mPEG-b-PJL and its functionalization into mPEG-b-PJL-OH. 3

Scheme S2. (A) Synthesis of thioketal linker (B) conjugation of thioketal linker to Doxorubicin and (C) conjugation of thioketal linker to Docetaxel.. 3

Scheme S3. Cleavage of thioketal bond in presence of reactive oxygen species (ROS) leading to the release of modified DOX and DTX structure. 4

Figure S1. ^{1}HNMR spectra of synthesized thioketal linker in $CDCl_3$ 4

Figure S2. ^{1}HNMR spectra of doxorubicin in DMSO-d_6. 5

Figure S3. ^{1}HNMR spectra of docetaxel in DMSO-d_6. 5

Figure S4. ^{1}HNMR spectra of mPEG-b-PJL-OH in $CDCl_3$. 6

Figure S5. 1HNMR spectra of PJL-DOX-DTX in DMSO-d6. 6

Figure S6. UV-Vis spectra of pure DOX, PJL-DOX-DTX micelles in milliQ water, and Ce6 in ethanol.. 7

Figure S7. HPLC traces of (A) doxorubicin (B) docetaxel, (C) chlorin e6 and (D) PJL-DOX-DTX. 7

Figure S8 Cleavage of thioketal linker in the presence of Ce6. 8

Figure S9. Cytotoxicity study of free DOX, DTX and PJL-DOX-DTX micelles with light expsoure before incubation on MDA-MB-231 cell lines for 48 h. 8

Figure S10. Cytotoxicity study of free DOX, DTX and PJL-DOX-DTX micelles at higher concentration on MDA-MB-231 cell lines for 48 h..9
References..9

Materials:

Poly(ethylene glycol) methyl ether (mPEG, Mn = 5.0 KDa), 1,5,7-triazabicyclo [4.4.0]dec-5-ene (TBD) (98%), mercaptopropionic acid (99%), dimethoxy-2-phenylacetophenone (99%), triethylamine (TEA) (≥99.5%), N-(3- dimethylaminopropyl)-N′-ethylcarbodiimide (EDC) (≥97%), 4-(dimethylamino) pyridine (DMAP) (≥99%), N-hydroxysuccinimide (NHS) (≥99.5%), HPLC grades methanol (≥99.9%), acetone (≥99.8%), deuterated dimethyl sulfoxide (DMSO-d6), deuterated chloroform ($CDCl_3$), THF, DMF, DCM, Diacetyldichlorofluorescein (DCFH-DA), Paraformaldehyde (PFA) and hexane were purchased from Sigma-Aldrich, Finland. Jasmine lactone (≥97%) was purchased from Lluch Essence, Spain. Doxorubicin (>99%) and Docetaxel (>99%) were purchased from LC Laboratories, USA. Chlorine e6 (Ce6) (≥90%) was purchased from Cayman Chemical, USA. Resazurin, 1,3-Diphenylisobenzofuran (DPBF) were purchased from TCI Europe Pvt. Ltd. VECTASHIELD (mounting medium with DAPI) was purchased from Vector Laboratories, Inc. Burlingame, CA.

Instruments:

Nuclear Magnetic Resonance (NMR) spectroscopy:

The chemical structure of polymers and conjugates were analysed by nuclear magnetic resonance (^{1}HNMR) spectroscopy on Bruker NMR 500 MHz (Bruker, Coventry, United Kingdom). DMSO-d6 andCDCl3 were used as solvents.

High Performance Liquid Chromatography (HPLC):

The absence of free DOX, Ce6 and DTX in the purified polymer-drug conjugate was confirmed by HPLC (Merck D-7000). The mobile phase comprises of water (pH 3 adjusted using phosphoric acid) and acetonitrile. The sample injection volume was 30 µl. The elution gradient had aqueous phase of 95% for the first 5 minutes, 40% for 6–8 min, 12% for 9–12 min, and then 95% upto 20 min with a flow rate of 1 ml/min. The Phenomenex Gemini C-18 column (5 µm, 4.6 x 150 mm) was employed for separation and drugs were detected at 233 nm.

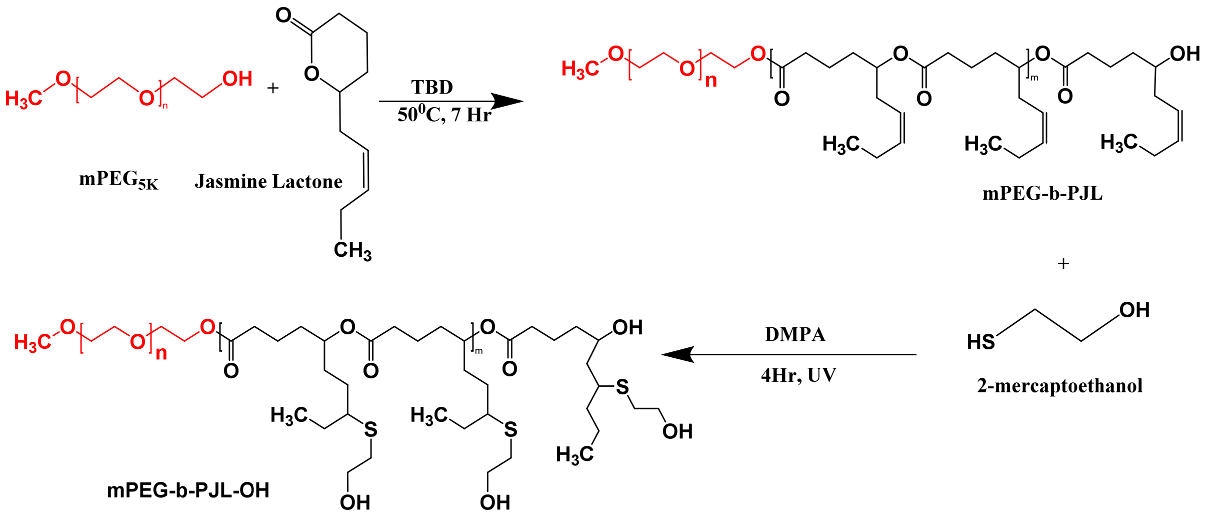

Scheme S1. Synthesis of mPEG-b-PJL and its functionalization into mPEG-b-PJL-OH. (1,5,7-triazabicyclo [4.4.0] dec-5-ene (TBD), Dimethoxy-2-phenylacetophenone (DMPA), UV cabinet fitted with a blacklight 368nm lamp (15W, Sylvania) and a stirrer.

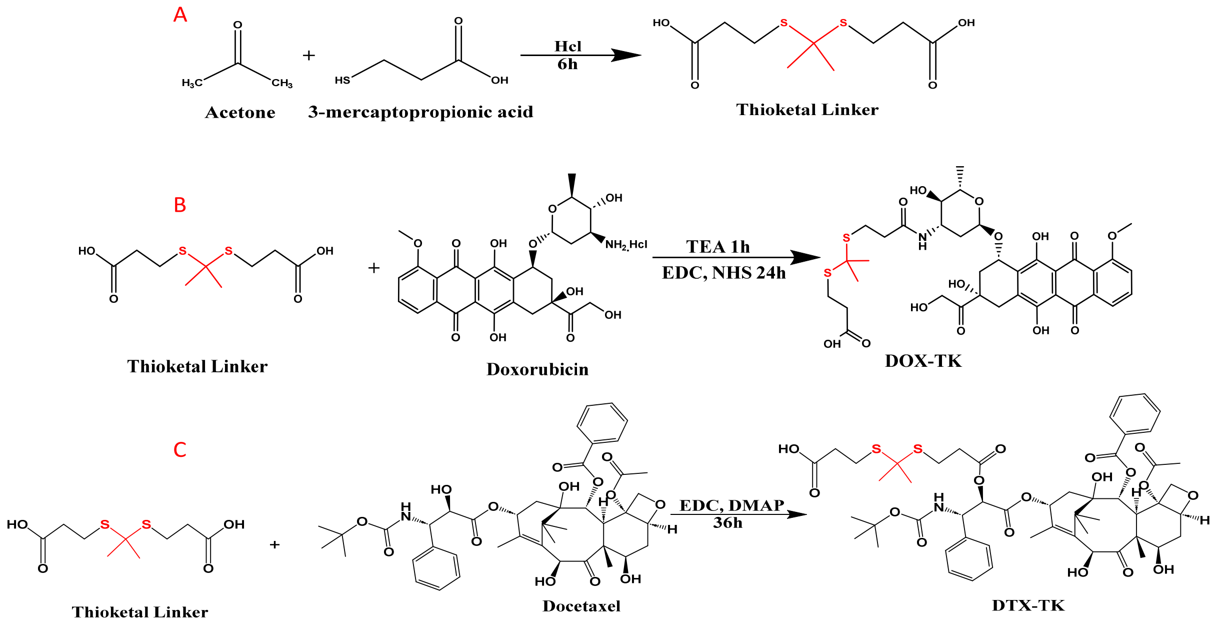

Scheme S2. (A) Synthesis of thioketal linker (B) conjugation of thioketal linker to Doxorubicin and (C) conjugation of thioketal linker to Docetaxel. Dry hydrogen chloride (Hcl), (N-(3-Dimethylaminopropyl)-N′-ethylcarbodiimide (EDC), 4-(Dimethylamino) pyridine (DMAP), N- Hydroxy succinimide (NHS), triethylamine (TEA).

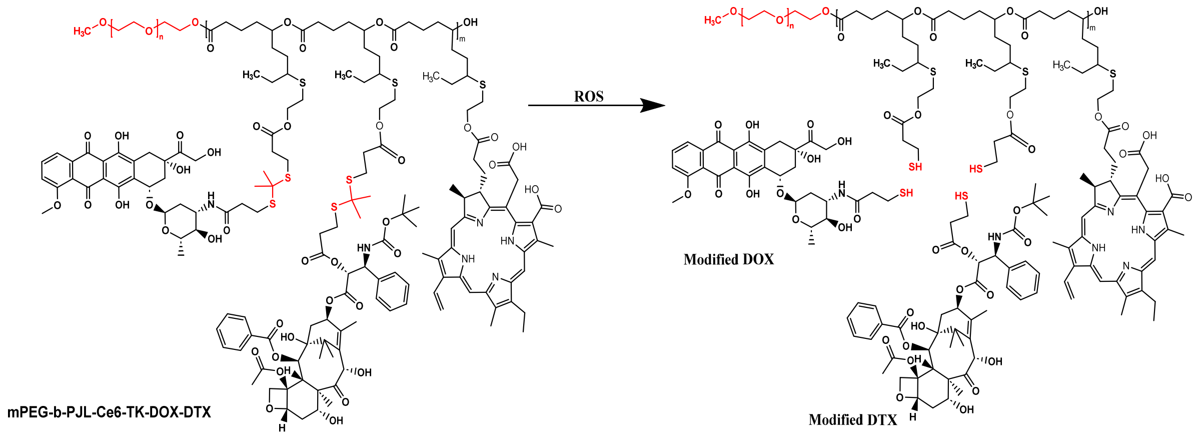

Scheme S3. Cleavage of thioketal bond in presence of reactive oxygen species (ROS) leading to the release of modified DOX and DTX structure [1].

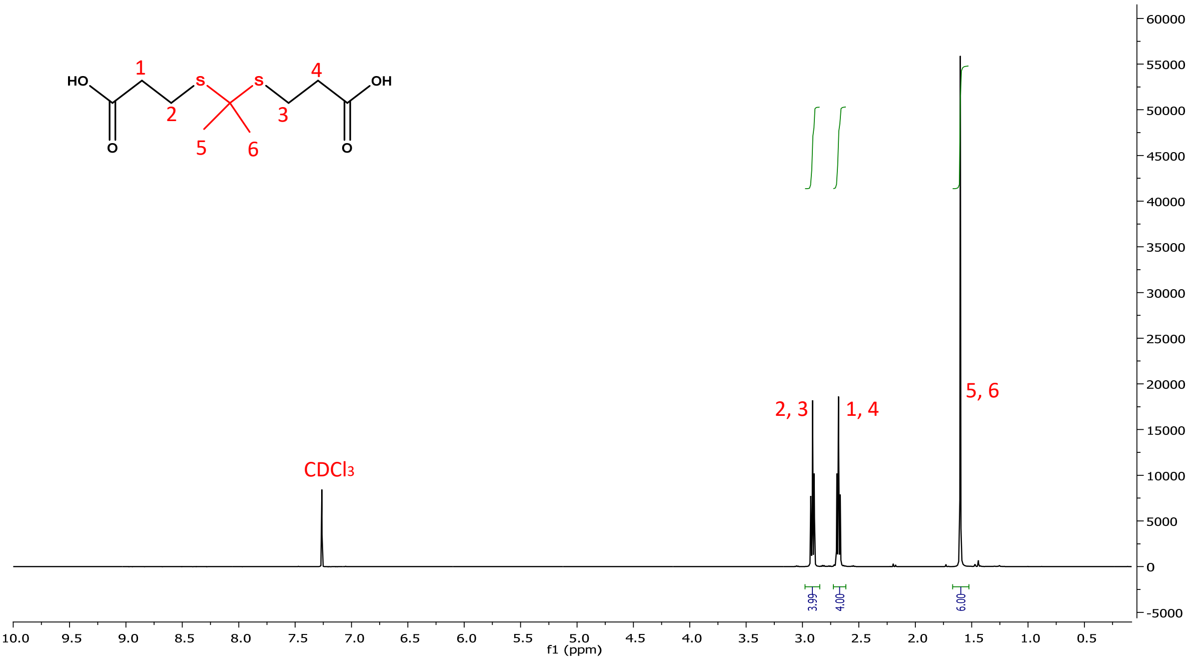

Figure S1. ^{1}HNMR spectra of synthesized thioketal linker in $CDCl_3$.

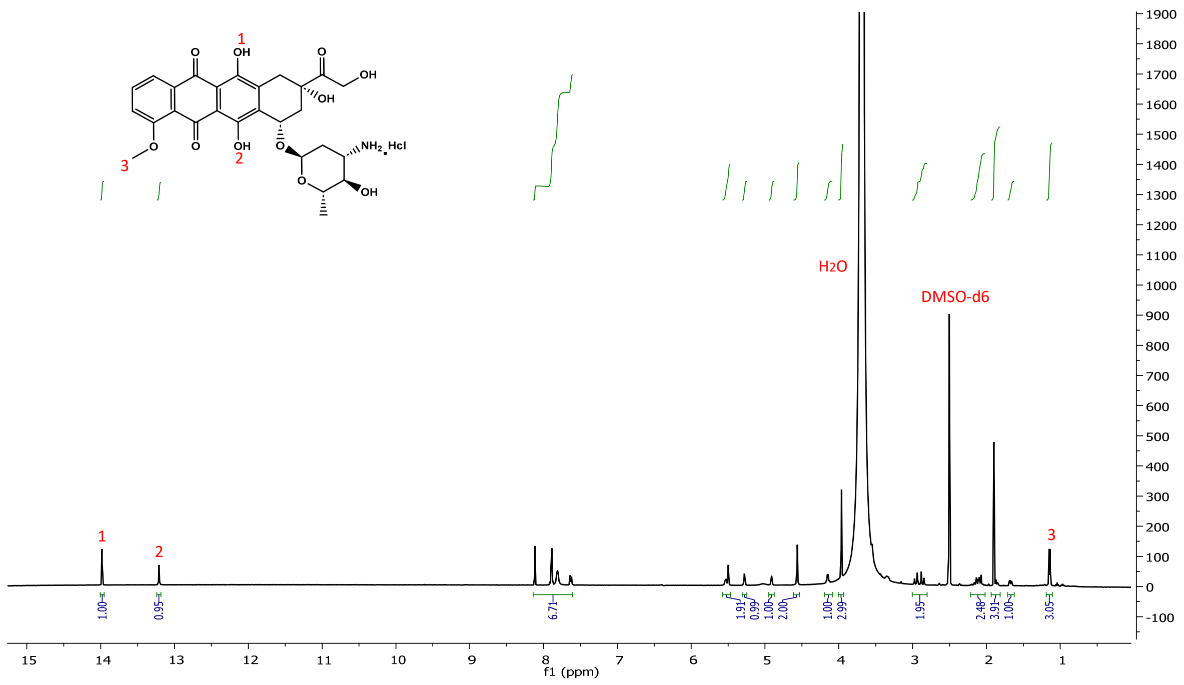

Figure S2. ^{1}HNMR spectra of doxorubicin in DMSO-d_6.

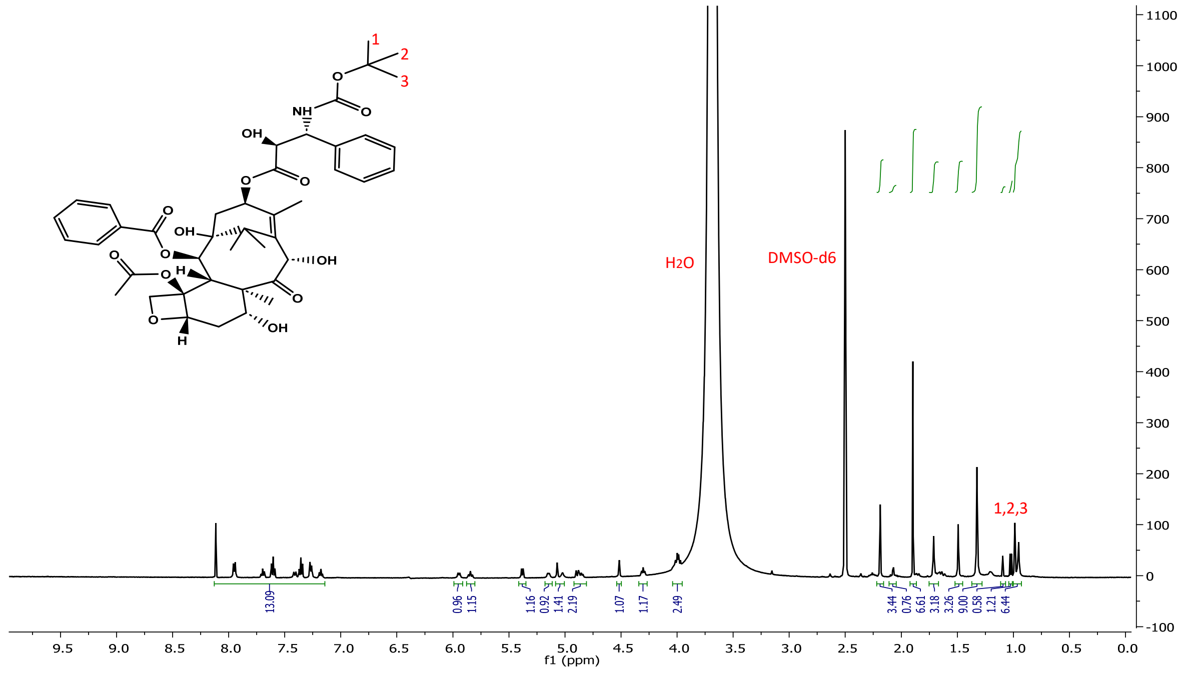

Figure S3. ^{1}HNMR spectra of docetaxel in DMSO-d_6.

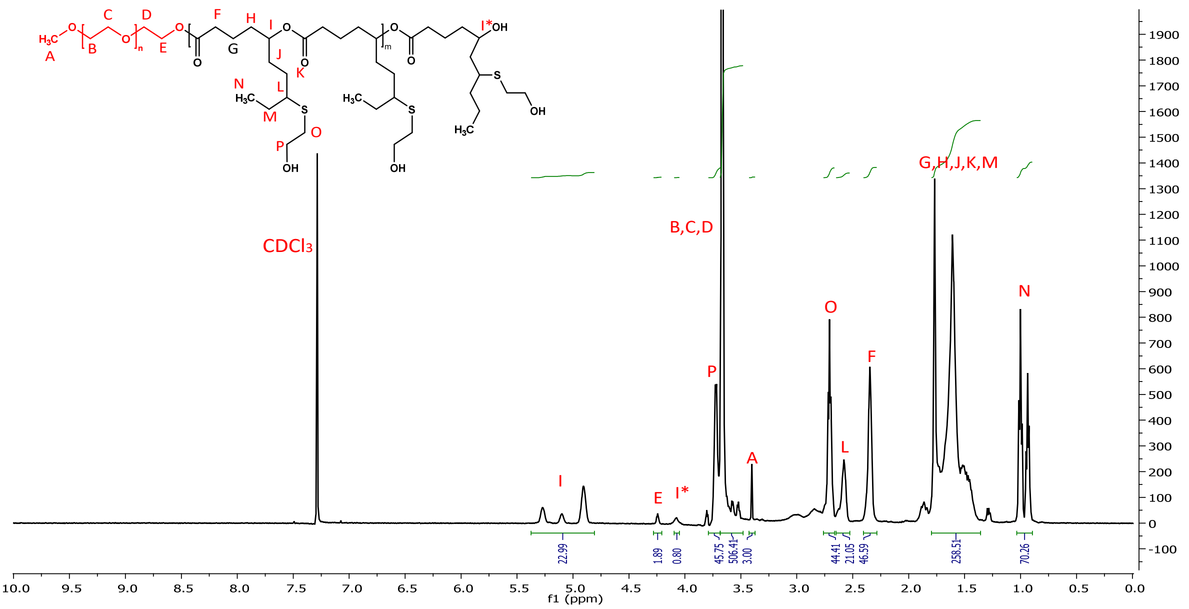

Figure S4. ^{1}HNMR spectra of mPEG-b-PJL-OH in $CDCl_3$. Molecular weight was calculated by comparing the protons at 4.9 ppm with the protons of mPEG at 3.3.

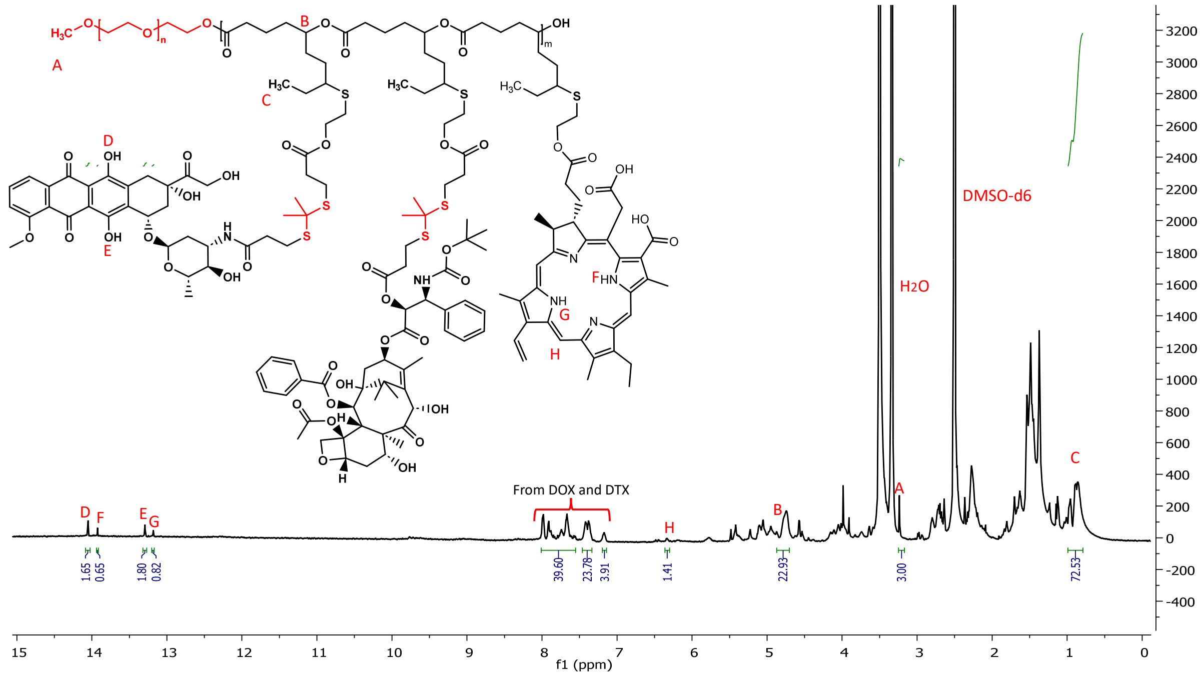

Figure S5. ^{1}HNMR spectra of PJL-DOX-DTX in DMSO-d6. The number of DOX, DTX and Ce6 molecule conjugated onto the polymer was found to be 2, 4 and 1 respectively.

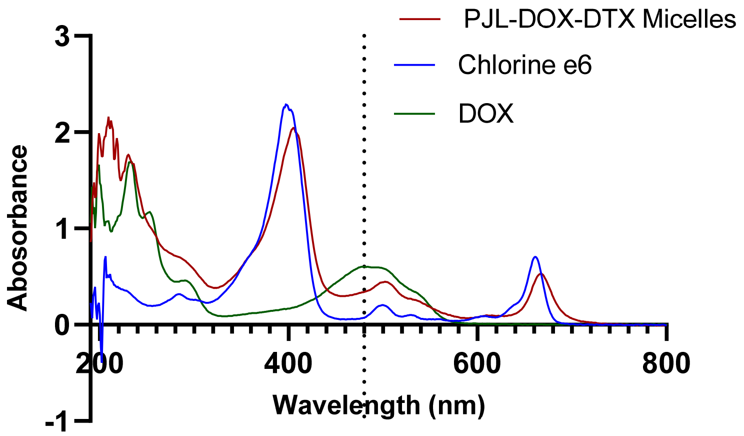

Figure S6. UV-Vis spectra of pure DOX, PJL-DOX-DTX micelles in milliQ water, and Ce6 in ethanol. The dotted line representing the λmax of DOX.

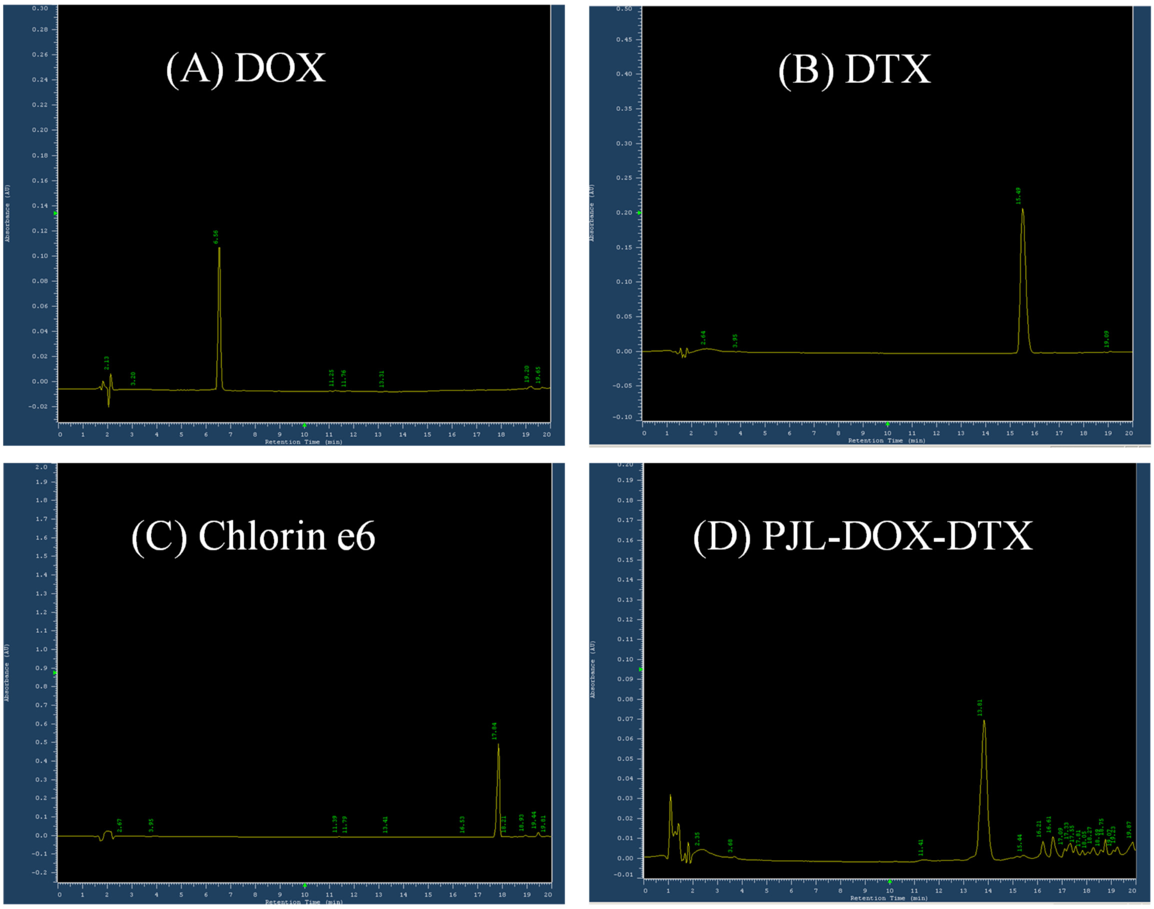

Figure S7. HPLC traces of (A) doxorubicin (B) docetaxel, (C) chlorin e6 and (D) PJL-DOX-DTX.

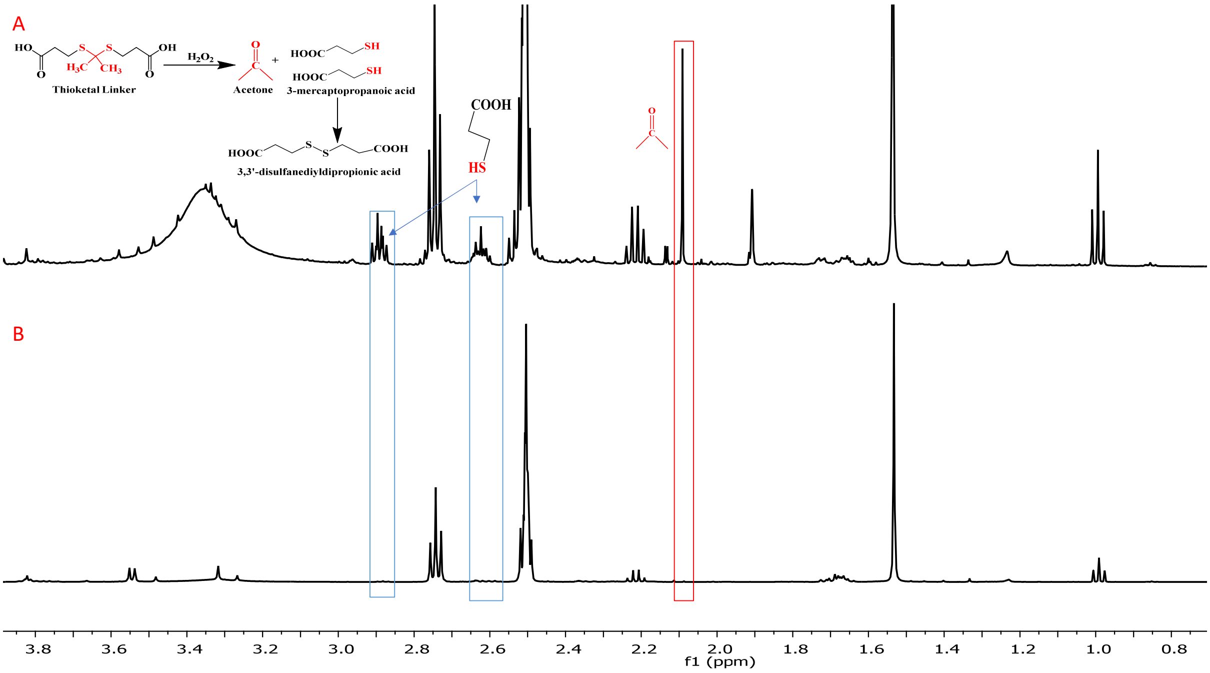

Figure S8. Cleavage of thioketal linker in the presence of Ce6. (A) ^{1}HNMR after irridiation detects the signals at 2.09ppm , 2.65ppm, and 2.9ppm, which belongs to the degraded product (acetone and 3-mercaptopropionic acid respectively) of thioketal linker. (B) ^{1}HNMR before irridiation.

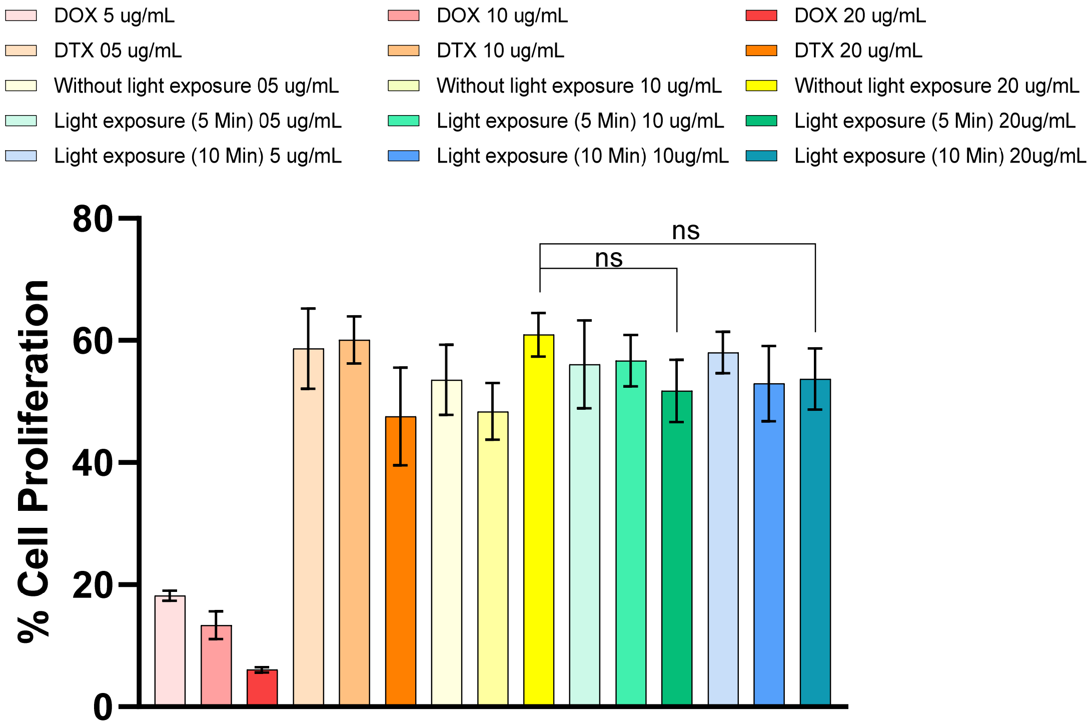

Figure S9. Cytotoxicity study of free DOX, DTX and PJL-DOX-DTX micelles with light expsoure before incubation on MDA-MB-231 cell lines for 48 h. Micelles exposure to NIR light (for 5 and 10 min) was done in a glass vial.

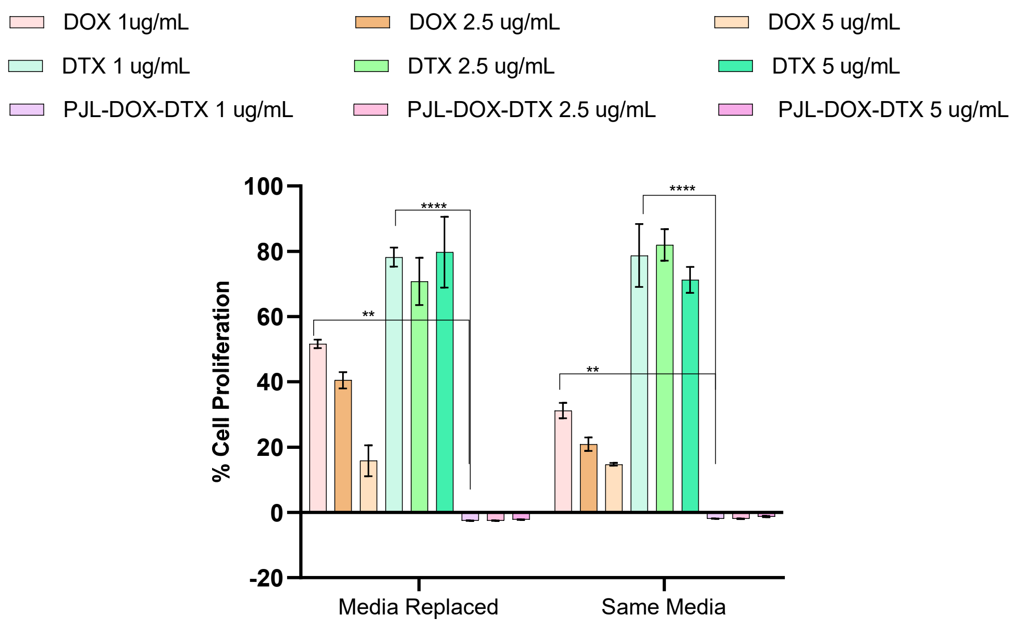

Figure S10. Cytotoxicity study of free DOX, DTX and PJL-DOX-DTX micelles at higher concentration on MDA-MB-231 cell lines for 48 h.

References

1 Ling, X. *et al.* Synthesis of a reactive oxygen species responsive heterobifunctional thioketal linker. *Tetrahedron letters* **56**, 5242-5244, doi:10.1016/j.tetlet.2015.07.059 (2015).
